# Successful Robotic Transabdominal Preperitoneal Approach for Recurrence of Anterior Mesh Plug Inguinal Hernia Repair: A Case Report

**DOI:** 10.7759/cureus.100971

**Published:** 2026-01-07

**Authors:** Masatsugu S Ishii, Toshikatsu Nitta, Yasuhiko Ueda, Akitada Sada, Ryuutarou Kubo, Atsuhiro Komiya, Takashi Ishibashi

**Affiliations:** 1 Division of Surgery, Gastroenterological Center, Shiroyama Hospital, Habikino, JPN; 2 Department of General and Gastroenterological Surgery, Osaka Medical and Pharmaceutical University Hospital, Takatsuki, JPN

**Keywords:** inguinal hernia, mesh plug, recurrence, robot, surgery, transabdominal preperitoneal

## Abstract

Recurrent inguinal hernia after mesh plug repair remains technically challenging, particularly due to distorted anatomy and fibrosis. Robotic surgery may offer advantages in such complex reoperative cases.

An 87-year-old male presented with recurrent right inguinal hernia 20 years after anterior mesh plug repair. Computed tomography demonstrated recurrent herniation with fibrotic tissue involvement. Robotic transabdominal preperitoneal (TAPP) repair using the da Vinci Xi system (Intuitive Surgical, Inc., Sunnyvale, CA) was successfully performed. The postoperative course was uneventful, and no recurrence was observed during nine months of follow-up.

This case demonstrates that robotic TAPP repair is a feasible and safe option for recurrent inguinal hernia following anterior mesh plug repair.

## Introduction

Inguinal hernia repair is among the most performed operations worldwide, with more than 20 million repairs performed annually [1]. Recurrence after inguinal hernia repair is a serious clinical problem, and the risk of recurrence ranges from 1% to 10% depending on the primary approach, surgeon’s technique, and follow-up period [2].

Some surgeons have noted the advantage of the robotic approach for more complex operations, such as recurrent inguinal hernia repair [3]. However, re-operative inguinal hernia repair is technically challenging due to disrupted tissue planes and adhesions, which have been well described in prior studies [3]. The robotic technology offers enhanced visualization, superior dexterity, and precision allied to wristed instruments to perform minimally invasive operations with finesse. This case report has been reported in line with the Surgical Case Report (SCARE) criteria [4]. Here, we report a case of a successful robotic transabdominal preperitoneal (TAPP) approach for the recurrence of anterior mesh plug inguinal hernia repair, with the patient remaining alive and free from recurrence nine months postoperatively.

## Case presentation

An 87-year-old male had undergone an inguinal hernia repair (mesh plug hernioplasty) 20 years earlier and had no ongoing medication related to that procedure. The patient reported progressive bulging in the right groin, consistent with a recurrent hernia. The laboratory findings are shown in Table 1. The patient had a history of atrial fibrillation and was receiving oral anticoagulant therapy. Anticoagulation was managed perioperatively according to institutional protocol, with temporary discontinuation prior to surgery. No perioperative thromboembolic or bleeding complications occurred.

**Table 1:** Preoperative blood examination results.

Parameter	Unit	Value	Reference range
White blood cell count (WBC)	×10³/μL	6.3	3.9 – 9.8
Red blood cell count (RBC)	×10⁶/μL	4.26	4.30 – 5.70
Hemoglobin (Hb)	g/dL	13.1	13.5 – 17.6
Hematocrit (Ht)	%	40.5	40.0 – 52.0
Platelet count (Plt)	×10⁴/μL	13.4	12.0 – 34.0
Aspartate aminotransferase (AST)	IU/L	22	10 – 40
Alanine aminotransferase (ALT)	IU/L	19	5 – 45
Alkaline phosphatase (ALP)	IU/L	72	110 – 340
Total bilirubin (T-Bil)	mg/dL	1.3	0.2 – 1.2
Total protein (TP)	g/dL	7	6.5 – 8.3
Albumin (Alb)	g/dL	3.7	3.8 – 5.2
Blood urea nitrogen (BUN)	mg/dL	21.6	8 – 20
Creatinine (Cr)	mg/dL	0.98	0.61 – 1.04
Sodium (Na)	mEq/L	141	135 – 147
Potassium (K)	mEq/L	4.1	3.3 – 5.0
Chloride (Cl)	mEq/L	106	98 – 108
C-reactive protein (CRP)	mg/dL	0.09	<0.3
Prothrombin time (PT)	sec	13.2	10.5 – 13.5
Prothrombin activity (PT)	%	69	70 – 130
Prothrombin time/international normalized ratio (PT/INR)	-	1.19	0.9 – 1.1
Activated partial thromboplastin time (APTT)	sec	42.7	25 – 40

Recurrent inguinal hernias after previous mesh-based repairs often present with distorted tissue planes that complicate re-operative dissection. Computed tomography revealed a right recurrent inguinal hernia with soft tissue involvement, and a mobile swelling was noted in the right inguinal region. Furthermore, no incarceration was observed, and a reduction was possible. With respect to hernia repair, we considered the anterior and inguinofemoral approaches for abdominal wall dissection to be difficult. Therefore, we planned a robot-assisted procedure.

The abdomen was insufflated to a pressure of 10 mmHg via a robotic trocar inserted through the umbilicus. Two additional 8-mm robotic trocars were placed in the right and left upper abdomen under direct visualization. Trocars of 8 mm were inserted through the right (R3) and left (R1) upper incisions to enable a 30° angle endoscope (12 mm robot trocar). A 30° endoscope was introduced through a 12-mm umbilical trocar, and two additional 8-mm robotic trocars were placed in the right and left upper abdomen under direct visualization. We performed targeting toward the inferior abdominal wall and viscera.

**Figure 1:**
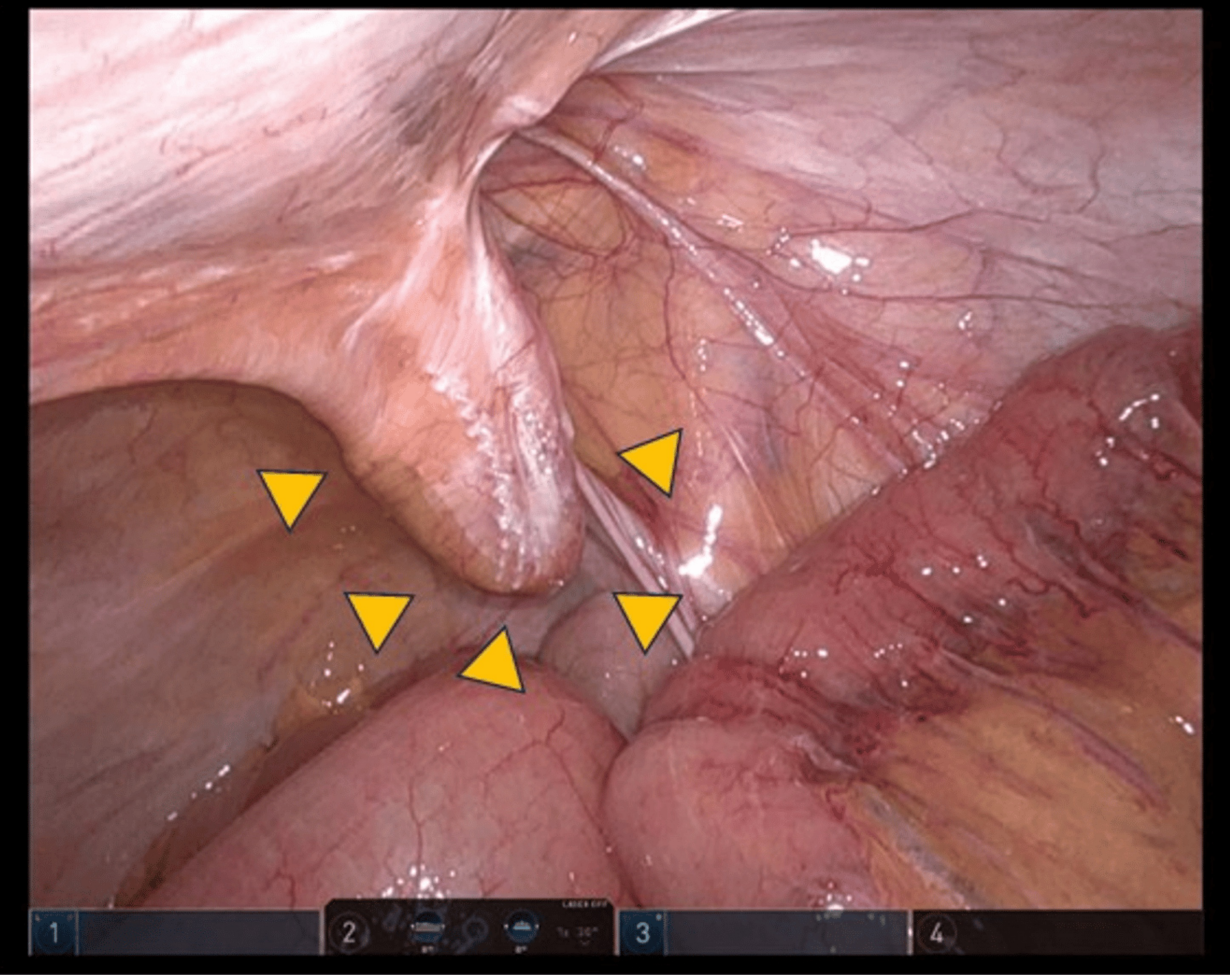
Recurrent right inguinal hernia. A recurrent right inguinal hernia with a mesh plug was confirmed (yellow arrow). Recurrent hernia repair was performed using the three arms of a bipolar fenestrated grasper, monopolar scissors, and a suture-cut needle driver. Preperitoneal dissection was initiated approximately 4 cm from the iliopubic tract, using a medial-to-lateral approach.

The patient was placed in a 0-15° head-down position. A robotic procedure using the da Vinci Xi surgical system (Intuitive Surgical, Inc., Sunnyvale, CA) was employed for all procedures using the retroperitoneal approach. A recurrent right inguinal hernia with a mesh plug (Figure 1) was observed. Recurrent hernia repair was performed using the three arms of a bipolar fenestrated grasper, monopolar scissors, and a suture-cut needle driver. Preperitoneal dissection was initiated approximately 4 cm from the iliopubic tract, using a medial-to-lateral approach.

**Figure 2:**
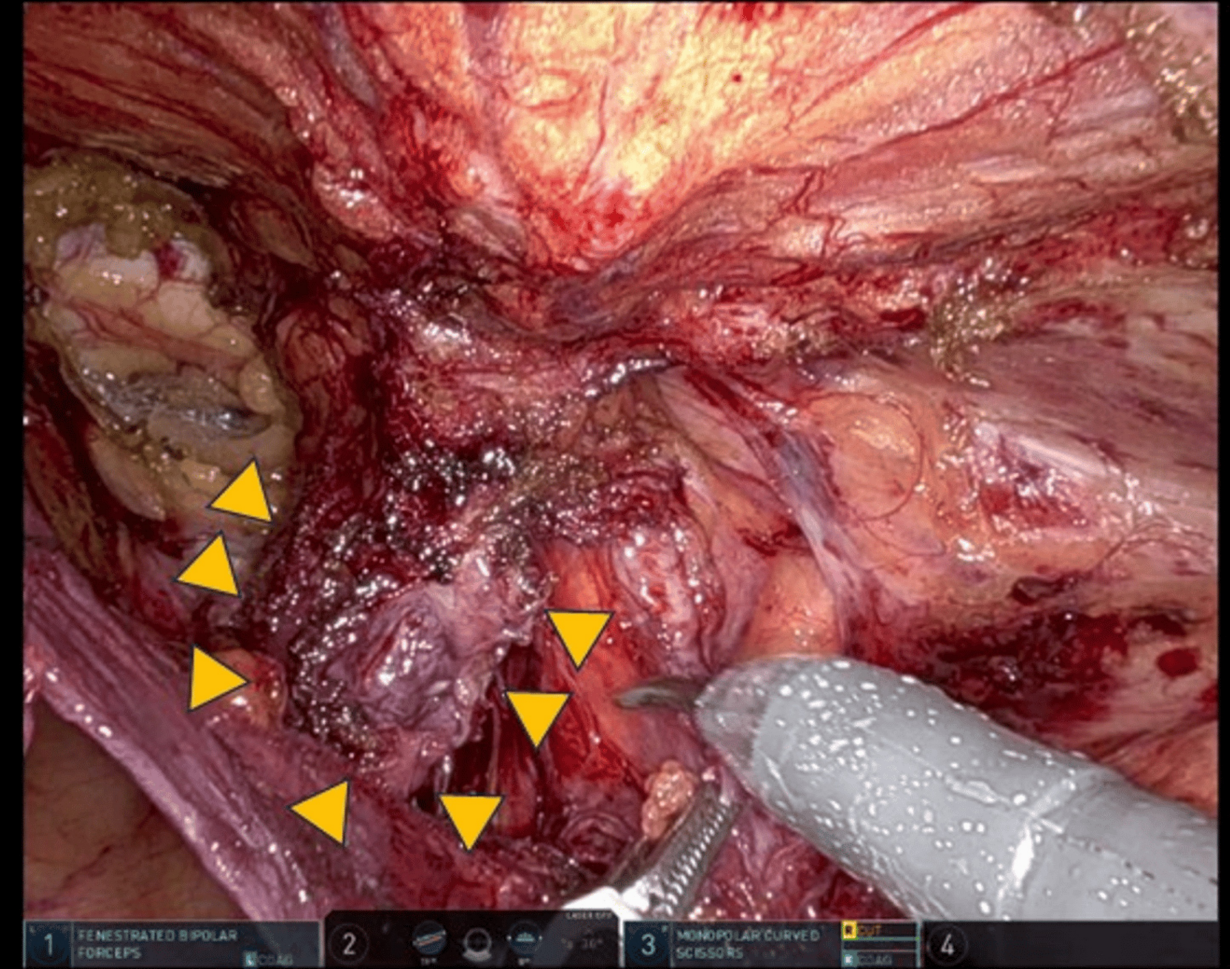
Recurrent right inguinal hernia. We dissected the preperitoneal space and kept it in contact with the adipose tissue in the abdominal wall. The medial and lateral spaces were connected after dissection of the intermediate fascia and parietal dissection of the visceral and cord planes (yellow arrow).

We dissected the preperitoneal space and kept it in contact with the adipose tissue in the abdominal wall. The medial and lateral spaces were connected after dissection of the intermediate fascia and parietal dissection of the visceral and cord planes (Figure 2).

Medial dissection was performed at least 2 cm medial to the rectus and 2 cm below the pubis to provide sufficient space for a hard Cooper’s ligament (CL) and for a large mesh. Dissection was performed 2 cm lateral to the ilium.

Here, the mesh plug changed to hard fibroblast tissue; therefore, it was difficult to dissect the avascular preperitoneal plane (Bogros’ plane). However, the peritoneum with a mesh plug was dissected as a peritoneal flap by using monopolar scissors, and we could make enough space to put in a hard mesh repair. Mesh placement was achieved by placing the mesh through a naval robot 12 mm trocar and introducing a prosthesis inside the abdominal cavity. A three-dimensional hard mesh (Bard 3D mesh, heavy type) was positioned to cover the entire myopectineal orifice with adequate overlap after revision hemostasis.

The type of mesh used may vary; however, its dimensions should be at least 15 × 12 cm. Larger meshes are recommended, particularly for larger direct defects or enlarged deep inguinal rings in inguinoscrotal hernias. The peritoneal flap was closed using running sutures. The total operative time was 156 minutes, and the estimated blood loss was 5 ml. No intraoperative complications occurred, and the postoperative course was good without complications. He had not complained of incisional and abdominal pain. On postoperative day one, he was started on a regular diet. The patient was then discharged on the third postoperative day. Subsequently, the patient showed no evidence of recurrence during the nine-month postoperative follow-up period.

## Discussion

The causes of inguinal hernia recurrence are multifactorial and include technical factors related to the initial repair, postoperative infection, and patient-related factors, such as impaired collagen metabolism and age-related tissue weakness. Early recurrence, which typically occurs within the first postoperative year, is commonly associated with technical factors related to the initial repair or postoperative infection. In contrast, late recurrence, developing several years after surgery, is more often attributed to progressive tissue weakness, impaired collagen metabolism, and age-related degeneration of the inguinal floor. He remains free of recurrence at nine months postoperatively, a period during which surgeon-related or infection-related failures typically become apparent and many years later comprise the smaller late group, which is commonly blamed for tissue failure. Late recurrence results from age-related defects in collagen metabolism, thinning of scar tissue, and continued inherent weakness of the inguinal floor. Although the benefits of laparoscopic inguinal hernia repair have been reported, this procedure is not widely performed, as extensive training in the posterior view with respect to the inguinofemoral anatomy and minimally invasive surgeons’ delicate skills are required, and it is easy to respond to various patterns using conventional laparoscopic surgery. Robotic technology has become an integral part of the minimally invasive urology and colorectal fields [5,6]. Their special characteristics include wristed movements, enhanced three-dimensional visualization, and excellent ergonomics for the surgeon, and they have been reported to be beneficial for inguinal hernia repair [7].

Currently, few comparisons between laparoscopic and robotic inguinal hernia repair are available. The robotic approach has been reported to be associated with a longer operative time than laparoscopic hernia repair [8]. However, in robotic inguinal hernia repair, mesh fixation is normally performed by suturing and not necessarily by endoscopic tacks. The peritoneal flap is closed using running sutures. Suturing for mesh fixation and peritoneal closure without endoscopic tacks could be associated with decreased chronic neurological pain and cost benefit [8].

Even if the recurrent form is unknown, adequate dissection and visualization of the previously operated area are possible with robotic surgery.

According to international groin hernia guidelines [9], the recommendation for recurrent hernia after anterior repair is posterior repair [9]. Furthermore, we have reported the robotic TAPP repair for recurrent hard inguinal hernia developed after Kugel hernioplasty, introducing the feasibility of our robotic surgical technique [10]. Several case reports and small series [10] have demonstrated the feasibility of robotic TAPP repair for recurrent inguinal hernia, particularly after prior posterior or preperitoneal approaches. These reports emphasize the advantages of robotic systems in complex re-operative settings, including enhanced three-dimensional visualization, articulated instrumentation, and improved ergonomics during meticulous dissection of scarred tissue planes. In contrast to many previously reported cases, the present patient had a recurrence after an anterior mesh plug repair, characterized by dense fibrotic transformation of the mesh plug and loss of the normal avascular preperitoneal plane. This scenario poses unique technical challenges and increases the risk of injury during conventional laparoscopic dissection. Our experience suggests that robotic TAPP repair allows controlled dissection and safe mesh placement even in such anatomically distorted conditions, supporting its potential role in selected cases of recurrent inguinal hernia following anterior repair. While we previously reported robotic TAPP repair for recurrent inguinal hernia following Kugel hernioplasty [11], the present case differs in several important aspects. In this patient, recurrence occurred after an anterior mesh plug repair, which is associated with dense fibrotic transformation of the mesh plug and severe distortion of the preperitoneal anatomy. This results in loss of the normal avascular plane and poses unique technical challenges distinct from those encountered after posterior or preperitoneal repairs. The present case, therefore, expands the applicability of robotic TAPP repair to recurrent inguinal hernias following anterior mesh plug hernioplasty, a commonly used primary technique, and provides additional technical insight into safe dissection and mesh placement in the presence of a hardened fibrotic mesh plug. Although this article is a case report that presents many limitations owing to a single-referred surgical group experience, our case shows that safe outcomes can be achieved by trained surgical groups familiar with the robotic platform that understand the posterior anatomy of the groin area.

## Conclusions

This case demonstrates that a robotic TAPP approach is feasible and safe for recurrent inguinal hernia after anterior mesh plug repair in selected patients. Further comparative studies are needed to clarify its potential advantages over conventional laparoscopic approaches.
